# The Pivotal Roles of the Epithelial Membrane Protein Family in Cancer Invasiveness and Metastasis

**DOI:** 10.3390/cancers11111620

**Published:** 2019-10-23

**Authors:** Mohammad Khusni B. Ahmat Amin, Akio Shimizu, Hisakazu Ogita

**Affiliations:** 1 Division of Molecular Medical Biochemistry, Department of Biochemistry and Molecular Biology, Shiga University of Medical Science, Otsu 520-2192, Japan; mdkhusni@belle.shiga-med.ac.jp (M.K.B.A.A.); shimizua@belle.shiga-med.ac.jp (A.S.); 2 Translational Research Unit, Department of International Collaborative Research, Molecular Neuroscience Research Center, Shiga University of Medical Science, Otsu 520-2192, Japan

**Keywords:** apoptosis, cancer progression, cell migration, cell proliferation, transmembrane protein

## Abstract

The members of the family of epithelial membrane proteins (EMPs), EMP1, EMP2, and EMP3, possess four putative transmembrane domain structures and are composed of approximately 160 amino acid residues. EMPs are encoded by the growth arrest-specific 3 (GAS3)/peripheral myelin protein 22 kDa (PMP22) gene family. The GAS3/PMP22 family members play roles in cell migration, growth, and differentiation. Evidence indicates an association of these molecules with cancer progression and metastasis. Each EMP has pro- and anti-metastatic functions that are likely involved in the complex mechanisms of cancer progression. We have recently demonstrated that the upregulation of EMP1 expression facilitates cancer cell migration and invasion through the activation of a small GTPase, Rac1. The inoculation of prostate cancer cells overexpressing EMP1 into nude mice leads to metastasis to the lymph nodes and lungs, indicating that EMP1 contributes to metastasis. Pro-metastatic properties of EMP2 and EMP3 have also been proposed. Thus, targeting EMPs may provide new insights into their clinical utility. Here, we highlight the important aspects of EMPs in cancer biology, particularly invasiveness and metastasis, and describe recent therapeutic approaches.

## 1. Introduction

**Figure 1:**
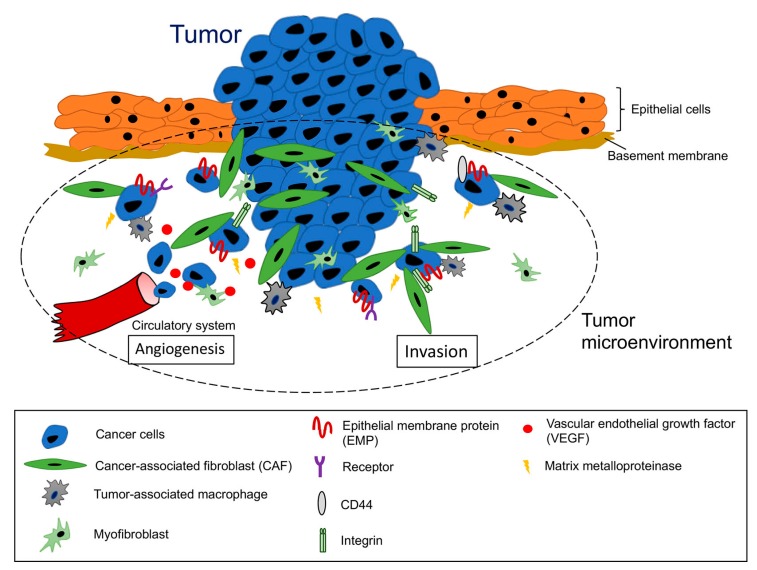
Schematic representation of the tumor microenvironment in cancer progression. When cancer cells derived from the original lesion travel across the basement membrane and invade the surrounding stromal tissue, they can interact with cells such as cancer-associated fibroblasts (CAFs), tumor-associated macrophages, and myofibroblasts to generate the tumor microenvironment. The characteristics of cancer cells are often affected by the tumor microenvironment, which increases the expression of cell surface proteins, including integrins and epithelial membrane proteins (EMPs), and several soluble factors, such as VEGF. These molecules associate with each other and may also regulate the cancer phenotype, such as invasiveness and angiogenic ability. Thus, understanding the tumor microenvironment through studies of cell surface molecules and their related molecules will likely contribute to the development of strategies designed to target cancer cells and to develop novel anti-cancer therapies.

Cancer metastasis describes the lethal process of the journey of cancer cells from the original site of tumor formation to other major organs, such as the kidney, liver, and lung [1,2]. The metastatic cascade proceeds through systematic steps, including invasion into the adjacent tissues, intravasation, circulation through blood or lymphatic vessels, anchoring at a secondary site, extravasation, and the formation of metastatic lesions in distant organs [3,4]. Supported by Paget’s theory [5], the tumor microenvironment, which harbors components such as cancer-associated fibroblasts (CAFs), myofibroblasts, endothelial cells, immune cells, and adipocytes, greatly influences the phenotypes of the metastatic cells [6,7,8,9]. CAFs reside in the stromal compartment and are associated with certain malignancies [10]. Most recently, the direct interaction of cancer cells with CAFs has been found to enhance the clonogenic growth and migration of pancreatic ductal adenocarcinoma cells [11]. The transition of cancer cells from a quiescent state to uncontrolled growth is driven by alterations of their local environment, which depends on stromal support [12,13]. The cross-talk between invasive and metastatic cancer cells and stromal tissue components is illustrated in Figure 1. Further, the outgrowth of latent breast carcinoma cells into the extracellular matrix (ECM) to colonize the lung is induced by specific ECM components, such as tenascin C and periostin [14,15].

The local environment surrounding the tumor, called the tumor microenvironment, plays an important role in the complex biological process of cancer metastasis. Associated factors include the influence of growth factors and the functional role of stromal fibroblasts, particularly in the reciprocal response to CAFs [16,17]. Each factor fundamentally regulates tumor growth and progression. Moreover, the changes in the expression of metastasis-related genes, which usually occur during the interaction of cancer cells with the tumor microenvironment, become the key regulators of a metastatic event. The interactions promote the aggressive phenotypes of certain cancers, such as those of the bladder, endometrium, lung, urinary bladder, and breast cancer through programming the epithelial-mesenchymal transition (EMT) [18,19,20,21].

We have recently revealed that the expression of a transmembrane protein, epithelial membrane protein 1 (EMP1), is upregulated in co-cultures of human prostate cancer cells and prostate stromal cells [22]. This co-culture system was utilized to mimic a tumor microenvironment. In an animal model, EMP1 significantly enhances the migration of cancer cells and the formation of metastatic lesions in the lymph nodes, lungs, or both. Increased EMP1 expression in patients with prostate cancer occurs at the invasive front and is associated with malignancy. In this context, we highlight in this review the functional aspects of the EMP family members EMP1, EMP2, and EMP3 related to their pathophysiology, particularly where associated with cancer metastasis. EMPs belong to the growth arrest-specific 3 (GAS3)/peripheral myelin protein 22 kDa (PMP22) gene family. The family members possess topologies similar to those of members of the tetraspanin superfamily, also known as the transmembrane 4 superfamily (TM4SF) [23]. TM4SF is comprised of the connexin, tetraspanin, claudin, pannexin, and GAS3/PMP22 families. EMPs lack sequence similarities and key structural features characteristic of the tetraspanins, which contain four to eight cysteine residues in the second extracellular domain to form disulfide bonds with a highly conserved Cys–Cys–Gly motif [24,25]. There is an increasing number of studies focused on EMPs, which have certain physiological functions. Moreover, in the pathological regard, the abnormal expression of EMPs in cancer cells may indicate a connection with cancer progression. Despite insights into the biological and physiological roles of EMPs, detailed knowledge of their effects at the molecular level associated with pathological conditions remains elusive. In the last part of this review, we consider future therapeutic approaches designed to improve the prognosis of cancers according to the molecular properties of EMPs.

## 2. The Structures, Expressions, and Functions of EMPs

**Figure 2:**
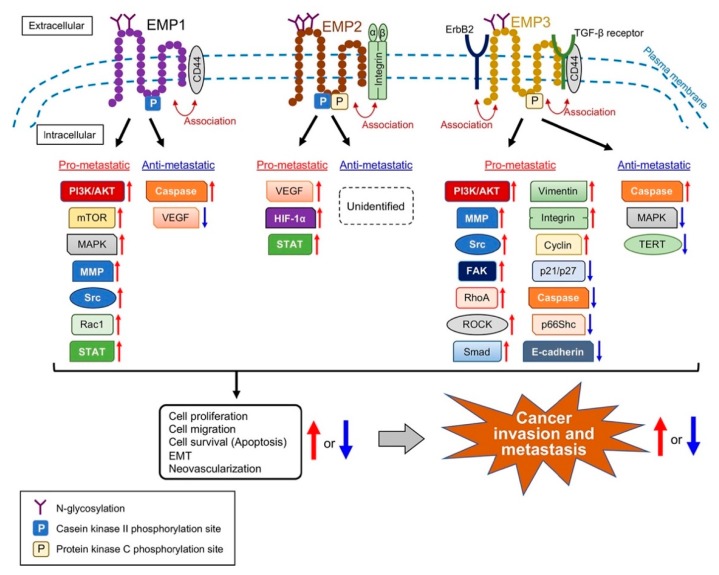
Structural difference between EMPs, and signal transduction molecules of which expression and/or activation are affected by EMPs in the regulation of cancer invasion and metastasis. The number and position of N-glycosylation and the kinase-mediated phosphorylation are different between EMPs. Numerous signaling molecules and cell surface proteins are associated with the role of EMPs. They mediate the EMP-induced cellular functions, such as proliferation and migration, that regulate the invasiveness of cancer cells and subsequent metastatic events. Red arrows: increase or activation of the molecules or phenomena; blue arrows: decrease or inactivation of the molecules or phenomena.

EMPs are hydrophobic proteins of approximately 160 amino acid residues with four predicted transmembrane domains, two extracellular domains, and small intracellular regions (Figure 2). The molecular structures of EMP2 and EMP3 share features such as protein kinase C phosphorylation sites, but EMP1 lacks these sites. EMP1 and EMP2 contain casein kinase-2 phosphorylation sites. All of the EMPs have N-glycosylation sites. The amino acid sequence of human PMP22 is 35%, 39%, and 41% identical to those of human EMP1, EMP2, and EMP3, respectively [26]. PMP22 is mainly expressed in the peripheral nervous system, particularly in Schwann cells [27], and regulates cell proliferation and apoptotic activity [28,29]. Similar to EMPs, the atypical activity of PMP22 is correlated with metastatic progression in certain cancers [30,31,32]. The amino acid sequences of the predicted transmembrane domains are highly conserved among the EMP family members.

### 2.1. EMP1

Analysis of the HPA, GTEx, and FANTOM5 datasets shows that *EMP1* mRNA levels are high in the esophagus, followed by adipose tissue, and the gallbladder [33]. Further, the levels of EMP1 protein are highest in the stomach, but low in other tissues [34].

EMP1 is expressed during the development of the central and peripheral nervous systems, suggesting its association with neurogenesis [35]. Moreover, EMP1 is involved in the regulation of the cell cycle, cell-to-cell interactions, and cell death [26]. During the growth of fibroblasts and Schwann cells, the expression of EMP1 is inversely regulated by PMP22 [34,36], and EMP1 is a novel tight junction protein of the blood-brain barrier [37].

### 2.2. EMP2

EMP2 is most commonly expressed in the lung, skin, and esophagus and is least likely to be expressed in the pancreas and brain according to the Human Protein Atlas [38]. EMP2 is expressed in multiple structures of the eye, such as the cornea, ciliary body, and the epithelium of retina [39]. The transcript level of *EMP2* is elevated in the fetal lung and kidney, but not in the adult thymus and peripheral leukocytes [40].

EMP2 localizes within lipid raft domains and is likely to modulate the plasma membrane trafficking activities of integrins and major histocompatibility complex class 1 proteins [41,42]. EMP2 regulates the physiological function of several integrins during blastocyst implantation, cell division, adhesion, and migration [41,43,44,45]. In a retinal epithelial cell line, EMP2 induces intracellular collagen gel contraction through the activation of focal adhesion kinase (FAK) at two phosphorylation sites Tyr576 and Tyr577 [46]. EMP2 and the activation of FAK are closely associated with integrin β1 subunits [41,47,48]. The enhancement of cellular adhesion to collagens types I and IV, the increase in expression of α-smooth muscle actin, and the activation of F-actin, particularly at the cell periphery, are detected in retinal epithelial cells that overexpress EMP2. These findings indicate that EMP2 may function in the reorganization of the actin cytoskeleton and enhance cellular contractility and adhesion [46].

### 2.3. EMP3

EMP3 is expressed in numerous organs, as indicated in the Human Protein Atlas [49]. Unlike EMP2, the levels of *EMP3* mRNA are high in the adult peripheral leukocytes and relatively strong in the fetal lungs, liver, and kidneys [40]. However, lower levels are expressed in the fetal and adult brain.

Recently, published data have shown that EMP3 plays a role in the immune system. Overexpression of EMP3 in macrophages inhibits CD8^+^ cytotoxic T lymphocytes (CTLs) [50], which are involved in tumor progression, viral infection, and type IV allergy [51]. In contrast, knockdown of EMP3 expression enhances the induction of CTLs, secretion of interferon-γ, and the expression of IL-2 receptor α by CD8^+^ T cells. These findings represent the unique function of EMP3 in regulating the immune system via the inhibition of CTL induction.

## 3. Involvement of EMPs in Cancer Metastasis

After the discovery of the GAS3/PMP22 family members [52,53,54], numerous studies revealed the role of these membrane proteins in certain chronic diseases. These proteins possess pathological properties via interaction with their binding partners such as integrins. Integrins are cell surface receptors composed of α and β subunits, which influence the tumor microenvironment [55]. The EMP family members are expressed and implicated in numerous cancers, indicating their role in oncogenesis and tumor progression. Further, EMPs have become emerging molecules to be associated with cancer invasion and metastasis because of their abilities to regulate various signaling molecules (Figure 2). The interaction with the specific receptors of ECM, including integrins, can regulate a dramatic consequence at the molecular level with respect to tumor progression. Among the EMP family members, EMP2 is expressed in the endometrium as a complex with integrin αvβ3, which resides on the apical side of the luminal epithelium [44,56,57,58]. EMP3 enhances the expression of integrins, particularly α1, α2, α3, α5, α6, αV, and β1, to promote the proliferation and migration of urothelial carcinoma cells. The differential expression of EMPs in cancer cells may be associated with the regulation of certain structural and signaling molecules to perform fundamental effects on the multistep processes of cancer invasion and metastasis. However, the roles of EMPs in invasion and metastasis in diverse cancers are controversial.

### 3.1. EMP1

#### 3.1.1. Pro-Metastatic Roles of EMP1

EMP1 contributes to the metastasis of glioblastoma multiforme (GBM). The mRNA levels of *EMP1* are upregulated in GBM, compared with those in normal brain tissues, which may explain the poor clinical outcome [59,60]. Silencing EMP1 expression in glioblastoma cells inhibits their proliferation and invasiveness, as well as the expression of CD44 and matrix metalloprotease (MMP)-2 [59,60]. CD44 isoforms, which are translated from alternatively spliced mRNAs, stimulate the uncontrolled growth of glioma stem cells [61]. Signaling through the phosphatidyl inositol-3 kinase (PI3K)/AKT/mammalian target of rapamycin (mTOR) pathway and its downstream molecules is inhibited in the EMP1-ablated cells. Other consequences of the inhibition of dysregulated CD44 by silencing EMP1 expression include the suppression of the activities of transcription factors such as OCT4, SOX2, and Nanog [59]. Moreover, the implantation of EMP1-knockdown P3 glioma cells in athymic nude mice showed remarkably smaller tumor formations than that of control cells. In the mice implanted with EMP1-knockdown cells, the levels of a mitotic marker Ki-67 and MMPs were reduced, and overall survival was increased. Because the clinical outcome of this aggressive malignant brain tumor remains very poor and an estimated relative survival of 5 years after diagnosis is only 5.6% [62], the findings of biomarkers and pro- or anti-metastatic factors for GBM may be highly helpful to develop novel therapeutics and improve its prognosis.

In uveal melanoma, the most common intraocular malignancy, *EMP1* mRNA levels are higher in class 2 tumors than in class 1 tumors [63]. A recent study found that treatment with ICG-001, a molecule that can impair the WNT/β-catenin signaling pathway [64], has strong anti-cancer activity against uveal melanoma by inhibiting the expression of EMP1 and the mTOR and mitogen-activated protein kinase (MAPK) signaling pathways [65].

Tumor tissues from non-small cell lung cancer (NSCLC) patients contain higher levels of *EMP1* mRNA than the benign tissues [66]. Overexpression of EMP1 in lung cancer cells increases the rate of cell proliferation through the activation of the PI3K/AKT signaling pathway [67]. This also accelerates tumor progression in athymic mice. In addition, *EMP1* mRNA expression is correlated with the acquisition of gefitinib resistance in patients with NSCLC, and the correlation is independent of gefitinib-sensitizing mutations in epidermal growth factor receptor [68].

The significance of EMP1 expression has been reported in acute lymphoblastic leukemia (ALL). Increased expression of *EMP1* mRNA positively correlates with prednisolone-resistant leukemic cells [69]. Silencing EMP1 expression in prednisolone-resistant leukemic cell lines induces apoptosis, suppresses cell migration and adhesion, decreases the proliferation rate, and sensitizes cells to prednisolone. These phenomena are mediated by impaired activities of Src family kinases (Src, Yes, Fgr, Blk, and Hck) and their downstream effectors such as JNK, STAT3, STAT5, CREB, and NF-κB.

#### 3.1.2. Anti-Metastatic Roles of EMP1

In contrast to the above tumors, the anti-tumor effects of EMP1 are observed in nasopharyngeal cancer, in which EMP1 protein levels are increased in normal tissues compared with the tumor lesions [70]. These findings are supported by observations that patients with the negative or weak expression of EMP1 protein in malignant lesions have lower 5-year survival rates than those with higher EMP1 levels. Overexpression of EMP1 in nasopharyngeal carcinoma cells decreases cell viability, migration and invasion, and induces apoptosis.

EMP1 also suppresses the progression of gastrointestinal cancers. *EMP1* mRNA levels are low in 15 pairs of the esophageal cancer lesions compared with the adjacent normal areas, and overexpression of EMP1 in esophageal carcinoma cells inhibits their proliferation [71]. In agreement with this finding, cell cycle arrest at the S and G_1_ phases is prolonged in EMP1-overexpressed cells. Consistent with the tumor suppressive activity of EMP1 in esophageal cancer, EMP1 protein expression is lower in gastric cancer than in normal tissues [72]. In human colorectal cancer and normal adjacent colorectal tissues, EMP1 protein expression is significantly lower in the former (39.7% tissues positive) than the latter (90.3% tissues positive) [73]. Patients with high levels of EMP1 experience a significantly better survival rate than those with low levels of EMP1. Moreover, overexpression of EMP1 reduces the proliferation rate of colorectal adenocarcinoma cells, induces early apoptosis with the augmented caspase-9 level, and significantly diminishes cell migration and invasion.

EMP1 protein expression is reduced in high-grade serous ovarian cancer, compared with benign ovarian tumors [74]. The research group seems to consider that EMP1 may negatively regulate the progression of ovarian cancer and decrease the severity of the cancer, although the detailed evidence is not provided.

#### 3.1.3. Opposing Roles of EMP1 in Cancer Invasiveness and Metastasis in the Same Type of Cancer

The functions of EMP1 in breast and prostate cancers are reported to be controversial [22,75,76,77]. The reason for the opposing activities of EMP1 in cancer progression in these types of cancer should be determined in the future. Further, the exact mechanism of EMP1-mediated signal transduction pathways in the regulation of cancer metastasis is largely unanswered. EMP1 serves as a reliable marker that distinguishes the two most common histological types of breast cancer: invasive ductal and lobular carcinomas [75]. The mRNA level of *EMP1* is significantly higher in the latter (93.1%) than the former (16.3%). Lobular tumors often resist neoadjuvant therapy [78], and patients with these tumors experience a lower survival rate than those with ductal tumors [79,80]. These data may suggest the tumor-progressive function of EMP1. On the other hand, reduced protein expression of EMP1 is associated with a poor survival rate [76], suggesting that EMP1 acts as a tumor suppressor. Overexpression of EMP1 in breast cancer cells inhibits proliferation through reducing the expression of vascular endothelial growth factor (VEGF)-C, and attenuates cell migration and invasion. 

**Figure 3:**
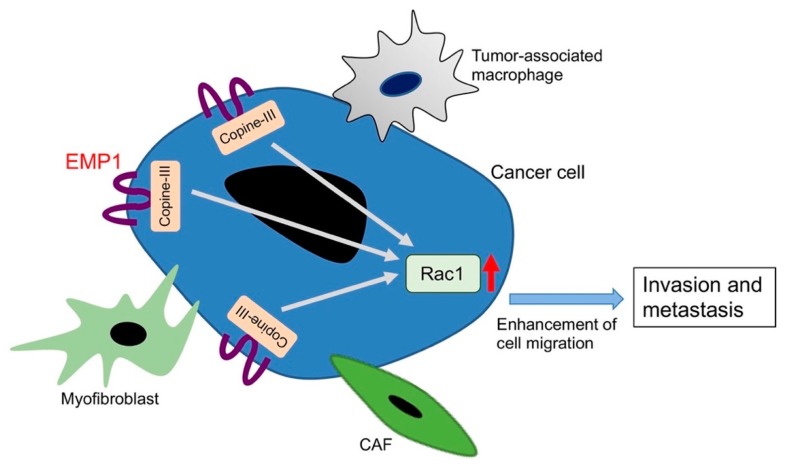
A model for the promotion of cancer invasion and metastasis by EMP1. Interaction of cancer cells with stromal cells, such as CAFs, myofibroblasts, and tumor-associated macrophages, upregulates the expression of EMP1, which activates Rac1 through the copine-III-mediated intracellular signal transduction. This enhances cancer cell migration to promote cancer invasion and metastasis. Red arrow: activation of Rac1.

Similar to breast cancer, the opposing roles of EMP1 in cancer invasion and metastasis operate in prostate cancer. We have recently found that EMP1 enhances the progression of prostate cancer in vitro and in vivo, and that the mRNA and protein levels of EMP1 are upregulated in co-cultures of human prostate cancer and stromal cells [22]. *EMP1* mRNA levels are lower in low grade cancer cell lines. Overexpression of EMP1 in LNCaP prostate cancer cells enhances cell migration and invasion, similar to the findings for MCF7 breast cancer cells and Caco2 colon adenocarcinoma cells. The inoculation of LNCaP cells overexpressing EMP1 in the prostate glands of nude mice promotes tumor metastasis to the lymph nodes and lungs, while control LNCaP cells do not metastasize. The sizes of the primary tumor in the prostate glands of the engrafted mice are identical between control and EMP1-overexpressed LNCaP cells, suggesting that EMP1 does not affect the proliferation of prostate cancer cells. Further, we successfully explored the mechanism of EMP1-induced cancer metastasis. The direct binding of copine-III to the intracellular region of EMP1 is critically involved in cancer invasion. The binding activates the signaling cascade of a tyrosine kinase Src, a Rac guanine nucleotide exchange factor Vav2, and a small GTPase Rac1, leading to enhanced cell migration and invasiveness. The similar effects of EMP1 are also observed in other types of cancer cell lines, MCF7 breast cancer cells and Caco2 colon cancer cells. EMP1 expression is significantly increased in the prostate cancer samples with higher Gleason scores, compared with those with lower scores. Our model of EMP1 function in cancer cells is displayed in Figure 3. In contrast, others uncovered the potential role of EMP1 as a suppressor of prostate cancer [77]. In this study, the levels of EMP1 protein are higher in normal tissues than in prostate tumors. Overexpression of EMP1 in PC3 prostate cancer cells significantly decreases cell migration and invasiveness. Thus, future studies are required to explain the discrepancy of EMP1 functions in breast and prostate cancers.

The summarized information about the pro- and anti-metastatic roles of EMP1 in cancers is shown in Table 1.

### 3.2. EMP2

#### 3.2.1. Pro-Metastatic Roles of EMP2

Studies on gene expression profiling elucidate that *EMP2* mRNA expression is upregulated in GBM [81,82]. The inoculation of GBM cells overexpressing EMP2 into nude mice accelerates tumor formation, mainly through the growing vasculature of intracranial tumors [83,84]. EMP2 and integrin αvβ3 interact in GBM cells to reciprocally stimulate their expression [83], which can augment cell migration and invasion through Src activation. STAT3 also enhances EMP2 expression and induces glioma-initiating cells to cause the progression and recurrence of GBM [85].

Transcriptional profiling analyses detected the dysregulation of *EMP2* mRNA expression in breast cancers [86,87,88,89]. EMP2 levels are significantly increased during the progression from non-malignant glandular to ductal carcinoma in situ, invasive ductal carcinoma, and lymph node metastasis [90], suggesting the potential role of EMP2 in the late stage of breast cancer in association with tumor invasion and metastasis [91]. *EMP2* mRNA expression might be negatively regulated by breast cancer-related receptors (estrogen receptor, progesterone receptor, and ErbB2), because the expression is relatively high in the triple-negative breast cancer cells [92]. The level of EMP2 protein is positively associated with cancer progression, which is supported by findings that EMP2 is expressed in 67% of lymph node metastases and is linked with lymphovascular invasion [90]. Recently, EMP2 protein seems to have been identified as a novel biomarker to effectively capture circulating tumor cells (CTCs) in blood samples from patients with breast cancer [93]. In addition, CTCs are more effectively detected in the samples from the pulmonary vein than the peripheral vein, even in the early stage of the cancer [94]. Because CTCs play a role in the development of cancer metastasis [95,96], earlier and effective detection of them is important.

In gynecologic cancers, approximately 70% of serous and endometrioid ovarian tumors express higher levels of EMP2 protein [97]. The protein level of EMP2 positively correlates with the progression of endometrial cancer [98,99,100], gradually increasing in the order benign, hyperplasia, atypical hyperplasia, and endometrial adenocarcinoma [101]. EMP2 mediates angiogenesis in endometrial cancer by regulating the expression of VEGF and hypoxia-induced transcription factors-1α through Src [99]. These findings suggest that EMP2 may serve as a potential candidate for pharmacological treatment of gynecologic cancers.

#### 3.2.2. Anti-Metastatic Roles of EMP2

EMP2 possesses anti-tumor properties by suppressing the growth of nasopharyngeal carcinoma cells [102]. EMP2 protein is expressed at low levels or is undetectable in most high-grade tumors (grades III and IV) in nasopharynx cancer. The clinical outcome of nasopharynx cancer is significantly worse for patients who do not detectably express EMP2. Ectopic expression of EMP2 in nasopharynx cancer cells in vitro impairs cell growth, enhances the efficiency of radiotherapy, induces cell cycle arrest at S phase, and increases apoptosis at both early and late stages. 

Similar to nasopharyngeal cancer, EMP2 has been discovered to act as a novel biomarker to suppress cutaneous melanoma [103]. The apoptosis in cultured melanoma cells is significantly induced by EMP2. EMP2 also functions as a tumor suppressor in urothelial carcinoma [104]. EMP2 protein expression inversely correlates with the histologic grades of uroepithelial cancer cells, and overexpression of EMP2 impairs cancer cell proliferation and inhibits tumor development.

The summarized information about the pro- and anti-metastatic roles of EMP2 in cancers is shown in Table 2.

### 3.3. EMP3

#### 3.3.1. Pro-Metastatic Roles of EMP3

EMP3 expression is upregulated to promote the progression of brain tumors [105,106,107], particularly in the mesenchymal subtype [107,108,109]. The expression of EMP3 and CD44 in GBM is relatively parallel [109]. CD44 is a biomarker for the mesenchymal subtype of GBM and is significantly related to unfavorable prognosis of patients with GBM [61,110]. In patients above age 50, high expression of *EMP3* mRNA is markedly associated with poor prognosis [108,111]. The overall survival rates of GBM patients with high EMP3 levels are reduced compared with those with low EMP3 levels [112]. Depletion of EMP3 protein attenuates proliferation and colony formation of GBM cells and induces apoptosis [109]. The interaction between EMP3 and transforming growth factor (TGF)-β receptor 2 was discovered in CD44-high GBM cells, leading to the activation of TGF-β receptor/Smad2/3 signaling. The inoculation of EMP3-depleted GBM cells in nude mice showed smaller tumor formation than that of EMP3-overexpressed cells. The expression of caspase-3, a marker of apoptosis, is upregulated in the tumor lysates derived from EMP3-depleted cells.

EMP3 contributes to the malignant phenotype of oral squamous carcinoma [113]. The expression of EMP3 is likely modulated by miR-765 in oral squamous carcinoma cells, and the depletion of EMP3 markedly stimulates the expression of p66Shc to impair the migratory activity of the cells.

Further, overall survival and the progression-free period are very poor in patients with gastric cancer with high levels of EMP3 [114]. EMP3 functions downstream of TWIST1/2 to induce EMT through the regulation of expression of an anti-EMT protein E-cadherin and an EMT marker vimentin. Similar to gastric cancer, EMP3 is highly expressed in HCC and negatively correlates with the degree of tumor differentiation [115]. Knockdown of EMP3 protein in hepatic cancer cells inhibits proliferation by arresting cell cycle at the G_1_ phase. The knockdown enhances the expression of cyclin-dependent kinase inhibitors p21 and p27, and reduces the levels of cyclins E and D1. The effects of high EMP3 expression on the malignant phenotype of HCC cells are associated with the increment of cell migration and invasion, as well as the proteolytic activities of MMP-9 and urokinase.

*EMP3* mRNA is significantly upregulated in lymph node metastases of breast carcinomas, and is related to the levels of ErbB2 expression [116,117]. EMP3 expression in breast cancer cells is negatively regulated by miR-765, which can bind to the 3′-UTR of *EMP3* mRNA. Recent bioinformatics analysis indicates that EMP3 contributes to the pathogenesis of endometrial cancer [118]. EMP3 may be associated with estrogen receptor α-mediated signaling pathways during the development of endometrial cancer.

The phenotypes of cells overexpressing EMP3 protein in upper urinary tract urothelial carcinoma are consistent with the conclusion that EMP3 confers the malignant characteristic. EMP3 and ErbB2 levels are high in high-grade urothelial carcinoma cells [119]. Cell proliferation and migratory activity are enhanced in urothelial carcinoma cells overexpressing EMP3, which increases signaling through the PI3K/AKT and FAK/Src pathways, leading to the activation of RhoA and ROCK1/2 and the upregulation of the expression of certain integrins. These findings highlight the importance of the EMP3−ErbB2 association as a potential indicator of progression and metastasis of upper urinary tract urothelial carcinoma.

#### 3.3.2. Anti-Metastatic Roles of EMP3

EMP3 protein expression negatively correlates with malignancy in esophageal squamous cell carcinoma [120]. The overexpression of EMP3 in esophageal cancer cells inhibits their growth and facilitates their death. The expression levels of EMP3 and telomerase reverse transcriptase inversely correlate in several esophageal cancer cell lines and tissue samples. The survival rate of patients with esophageal cancer after recurrence is significantly lower in patients with low levels of EMP3. 

Recently, EMP3 has been reported to suppress the progression of gallbladder cancer, and its low expression is strongly associated with poor prognosis [121]. Knockdown of EMP3 protein in gallbladder cancer cells that possess high levels of endogenous EMP3 increases proliferation, while overexpression of EMP3 inhibits migratory activity and promotes high apoptotic activity by increasing the expression of caspase-3 and caspase-9. The inoculation of gallbladder cancer cells overexpressing EMP3 into nude mice induces significantly smaller and lower weight tumors than that of EMP3-depleted cancer cells. The level of miR-663a is augmented in human gallbladder cancer tissues, and knockdown of this miRNA reciprocally increases EMP3 expression. Downregulation of EMP3 in gallbladder cancer activates the MAPK/ERK signaling pathway to promote cancer progression.

EMP3 suppresses NSCLC, consistent with findings that EMP3 protein levels are significantly lower in malignant lesions than in normal tissues [122]. The EMP3 levels in the tumor inversely correlate with the Ki67 expression, suggesting increased cell proliferation in the absence of EMP3 in malignant tumors.

The summarized information about the pro- and anti-metastatic roles of EMP3 in cancers is shown in Table 3.

### 3.4. PMP22

As described above, both PMP22 and EMPs belong to the GAS3/PMP22 family. Although mutations and loss of functions of PMP22 are related to demyelinating peripheral neuropathies [28,123], PMP22 is also reported to increase the aggressiveness of various types of cancers. According to the immunohistochemical and gene expression analyses of human breast cancer tissue samples, the mRNA level of *PMP22* is highly upregulated and contributes to poor overall survival and disease-free survival [32]. The *PMP22* mRNA is also increased in pancreatic ductal adenocarcinoma, compared with normal pancreatic tissues [124]. This pathological feature appears to be associated with the early development of malignancies. The ectopic expression of *PMP22* mRNA in tumor specimens of patients with gastric cancer is correlated with recurrence after perioperative chemotherapy, suggesting a crucial role of PMP22 in the resistance of chemotherapy [30]. This finding is supported by the experiments using xenograft model mice and PMP22-knockdown gastric cancer cells. The suppression of PMP22 expression in lung cancer cells by miR-29 impairs cell proliferation and invasion [125], and this result may suggest the involvement of PMP22 in progression of lung cancer.

## 4. Therapeutic Implications of EMPs

### 4.1. Development of Monoclonal Antibodies

The first therapeutic monoclonal antibody (mAb) rituximab was approved for clinical use in 1997 to treat non-Hodgkin’s lymphoma [126]. Rituximab treatment, which significantly improves the prognosis of patients with lymphoma [127], opened the era of biological cancer therapeutics, as evidenced by the numerous clinically-approved mAbs used to treat diverse cancers [128]. The current development of mAbs that target EMP family members to prevent cancer progression and metastasis is very challenging because of the clinicopathological heterogeneity and biological complexity of cancers. The anti-EMP2 recombinant bivalent antibody fragments (diabodies) reduce the aggressiveness of endometrial cancer cells [129]. These diabodies, KS49 and KS83, recognize human and mouse EMP2 peptides, respectively. Treatment of ovarian and endometrial cancer cells with the anti-EMP2 diabodies inhibits cell growth and increases apoptosis [97,129]. Moreover, treatment of breast cancer cells with anti-EMP2 IgG1 promotes cell death and inhibits cancer cell invasion [100]. In an animal model using BALB/c mice, anti-EMP2 treatment actually reduces the number of metastatic lesions generated by xenografted mammary gland tumor cells that express high levels of EMP2.

Despite current beneficial outcomes of pre-clinical trials in treatment with anti-EMP2, additional studies are required to assess acute and long-term adverse effects. Improvements in the pre-clinical implementation and the delivery method are necessary to optimize the treatment for future clinical use.

### 4.2. Perturbation of Protein−Protein Interactions

Targeting protein−protein interactions as a therapeutic intervention is very challenging, because the binding interfaces of proteins vary, thereby hampering efforts to predict the consequences of the targeting. Further studies are required to completely identify the effects on signal transduction networks induced by inhibitors of the relevant protein−protein interactions. If such studies are not well performed, the inhibitors may provoke the unexpected disturbance of the intricate balance inside the cell, leading to severe adverse effect.

Examples of studies designed to inhibit protein–protein interactions in order to prevent cancer progression are as follows: (1) An inhibitor of the interactions of bromodomain and extra-terminal family member BRD4 with other proteins in an incurable subtype of human squamous carcinoma [130]; (2) a competitor of the pro-survival protein Bcl-X_L_ [131]; (3) an inhibitor that interferes with binding of MDM2 to P53 [132]; and (4) an inhibitor that blocks the binding of VEGF to its receptor to prevent angiogenesis [133]. Further, as described above, we have found that copine-III binds to EMP1, which enhances signal transduction to influence the invasiveness of cancer cells [22]. Therefore, developing a specific inhibitor to block the binding of copine-III to EMP1 would terminate the invasiveness. The direct interaction between EMP1 and copine-III is mediated through a contiguous stretch of nine amino acid residues within the intracellular region of EMP1. To perturb the interaction, a potential inhibitor must enter the cell, possibly by adding a cell-penetrating sequence of the human immunodeficiency virus TAT protein. This technique was used to inhibit the activation of RhoA to prevent the uncontrollable proliferation of cancer cells via aberrant signaling through the VEGF-A/NRP1 signaling pathway [134].

## 5. Conclusions

The research reviewed here provides compelling evidence that members of the EMP family mediate cancer progression and metastasis. Further studies are clearly warranted to characterize in detail, the tumor progressive and suppressive activities of EMPs, to design treatment strategies in order to improve the prognosis of patients with cancer. Important examples presented here include the development of anti-EMP monoclonal antibodies and inhibitors of the binding of EMPs to their effectors.

## Figures and Tables

**Table 1:** Roles of EMP1 in cancer metastasis.

Type of Cancer	In Vitro Model		In Vivo Model		Patient Samples		Remarks
	mRNA	Protein	mRNA	Protein	mRNA	Protein	
**Pro-metastatic property**							
GBM	[59,60]	[59,60]		[59]	[59,60]	[59,60]	Promotion of cancer cell proliferation and invasion; Correlation with poor clinical outcome
Uveal melanoma	[65]	[65]	[65]		[63]		Possible relationship with a high risk for metastatic death
NSCLC	[66]	[66,67]		[67]	[68]	[67]	Increase in cancer cell proliferation; Impairment of drug sensitivity
ALL	[69]	[69]			[69]		Decrease in apoptosis; Increase in cancer cell migration, adhesion and proliferation; Impairment of drug sensitivity
**Anti-metastatic property**							
Nasopharyngeal cancer	[70]	[70]				[70]	Inhibition of cancer cell migration and invasion; Increase in apoptosis; Improvement of the patients’ survival rates
Esophageal cancer	[71]				[71]		Decrease in cancer cell proliferation
Gastric cancer	[72]	[72]				[72]	Correlation with reduced cancer invasion and metastasis and with elongation of the patients’ survival
Colorectal cancer	[73]	[73]				[73]	Decrease in cancer cell proliferation; Increase in apoptosis; Improvement of the patients’ survival rates
Ovarian cancer						[74]	Possible association with reduction of the severity of the cancer
**Paradoxical effect on metastasis**							
Breast cancer	[76]	[76]				[75,76]	Possible correlation with promotion of cancer invasion; Biomarker to distinguish the histological types of the cancer Inhibition of cancer cell migration, proliferation and invasion; Improvement of the patients’ survival rates
rostate cancer	[22,77]	[22,77]		[22]	[22]	[22,77]	Promotion of cancer cell migration, invasion and metastasis Inhibition of cancer cell migration and invasion

[ ] indicates the number of reference.

**Table 2:** Roles of EMP2 in cancer metastasis.

Type of Cancer	In Vitro Model		In Vivo Model		Patient Samples		Remarks
	mRNA	Protein	mRNA	Protein	mRNA	Protein	
**Pro-metastatic property**							
GBM	[84]	[83,84]		[83,84]	[82]	[81,83]	Promotion of cancer cell migration, invasion and angiogenesis
Breast cancer	[92]	[90,93]		[92]	[86,87,88,89,91]	[90,93]	Promotion of cancer cell invasion and metastasis
Ovarian cancer		[97]		[97]		[97]	Decrease in cancer cell death; Association with the malignant type of the cancer
Endometrial cancer	[99,100]	[98,99,100]		[98,99,100]		[101]	Promotion of angiogenesis; Correlation with cancer progression
**Anti-metastatic property**							
Nasopharyngeal cancer	[102]	[102]				[102]	Decrease in cancer cell growth; Enhancement of the sensitivity of radiotherapy; Improvement of the clinical outcome
Cutaneous melanoma	[103]	[103]				[103]	Increase in apoptosis
Urothelial cancer	[104]	[104]		[104]		[104]	Decrease in cancer cell proliferation

[ ] indicates the number of reference.

**Table 3:** Roles of EMP3 in cancer metastasis.

Type of Cancer	In Vitro Model		In Vivo Model		Patient Samples		Remarks
	mRNA	Protein	mRNA	Protein	mRNA	Protein	
**Pro-metastatic property**							
GBM	[109]	[109]		[109]	[105,106,107,108,109,111,112]	[112]	Increase in cancer cell proliferation; Decrease in apoptosis; Correlation with poor clinical outcome
Oral squamous cancer		[113]			[113]	[113]	Increase in cancer cell migration
Gastric cancer	[114]	[114]			[114]		Induction of EMT; Correlation with poor clinical outcome
Hepatocellular cancer		[115]		[115]		[115]	Promotion of cancer cell proliferation, migration and invasion; Negative correlation with tumor differentiation
Breast cancer	[116]	[116]			[116,117]	[116]	Promotion of cancer cell proliferation, invasion and metastasis
Endometrial cancer					[118]		Possible correlation with development of the cancer
Urothelial cancer	[119]	[119]			[119]	[119]	Increase in cancer cell proliferation and migration
**Anti-metastatic property**							
Esophageal cancer	[120]	[120]			[120]	[120]	Increase in cancer cell death; Improvement of the survival rate
Gallbladder cancer	[121]	[121]		[121]	[121]	[121]	Inhibition of cancer cell proliferation, migration and invasion; Improvement of the patients’ survival rates
NSCLC		[122]				[122]	Decrease in cancer cell proliferation

[ ] indicates the number of reference.
